# Interpreting Heterogeneity Beyond *I* ^2^ in Meta‐Analysis: Integrating Statistical Metrics With Clinical Judgement

**DOI:** 10.1111/jep.70601

**Published:** 2026-09-08

**Authors:** Matheus Hissa Lourenço Ferreira, Lucas Caseri Câmara, Francisco D'Paula Vitor Ferreira, Miércio Soares Melo, Rebeca Ferreira de Souza, Débora Regina de Aguiar, Samuel Izaias dos Santos Pereira, Nelson Carvas Junior

**Affiliations:** ^1^ Federal University of Minas Gerais (UFMG) Belo Horizonte Minas Gerais Brazil; ^2^ Brazilian Society of Endocrinology and Metabolism in Sports and Exercise, Department of Specialisation in Clinical Anabolism, College of Governance Engineering and Education of São Paulo (FGE‐SP) São Paulo São Paulo Brazil; ^3^ State University of Goiás (UEG) Itumbiara Goiás Brazil; ^4^ Federal University of São Paulo (UNIFESP) São Paulo São Paulo Brazil; ^5^ University of Buenos Aires (UBA) Buenos Aires Buenos Aires Argentina; ^6^ Paulista University (UNIP), São Paulo, São Paulo, Brazil; Department of Postgraduate Studies in Evidence‐Based Health Federal University of São Paulo (UNIFESP), São Paulo São Paulo Brazil

**Keywords:** clinical decision‐making, evidence‐based medicine, heterogeneity, meta‐analysis as topic, reproducibility of results

Meta‐analysis is a central tool for evidence synthesis in health research, influencing clinical guidelines, public policies, and decision‐making [1, 2]. Assessing heterogeneity among studies is essential for interpreting results. The *I* ^2^ statistic is widely used and often treated as a standalone decision‐making criterion to judge consistency and guide methodological choices [3]. However, this practice oversimplifies a complex phenomenon and may lead to inappropriate statistical and clinical interpretations, particularly when fixed thresholds (e.g., *I* ^2^ ≥ 50%) are used to define ‘substantial heterogeneity’ or to determine whether results should be pooled [1, 4, 5]. This article questions the use of *I* ^2^ as an isolated criterion and advocates a multidimensional and contextually grounded interpretation of heterogeneity.

Heterogeneity in meta‐analyses is multidimensional and cannot be adequately characterised by a single metric [6]. Systematic reviews inevitably include clinical (differences in populations, interventions, comparators, and outcomes), methodological (variations in design, measurement, and risk of bias), or statistical heterogeneity (observed effects differing beyond sampling error) [6, 7, 8]. Statistical heterogeneity largely reflects underlying clinical and methodological diversity. Clinical differences may indicate genuine variation in effects across contexts, whereas methodological differences may generate variability in observed estimates without implying true effect modification [7, 9]. Recognising this multidimensional structure is essential to avoid simplistic conclusions based solely on statistical indicators [6, 7, 8, 9].

Statistical heterogeneity arises when observed study effects differ more than expected from sampling error [7]. It is detectable in estimated effects but does not automatically imply true differences across studies [7, 8]. Cochran's *Q* test assesses whether observed variability is compatible with chance alone, but evaluates only the presence—not the magnitude or relevance—of heterogeneity [7, 8]. Its limitations are well known: low power with few studies and excessive sensitivity when many large studies are included, making results dependent on study number [7, 8]. Non‐significant results (often *p* > 0.10) indicate only the absence of statistically detectable heterogeneity [6, 7, 8, 10].

The *I* ^2^ statistic was proposed to express the proportion of total variability in estimated effects attributable to inconsistency beyond sampling error, rather than to sampling error alone [7, 8]. Unlike the *Q* test, *I* ^2^ does not depend on a statistical significance level and is widely used to describe the degree of inconsistency in meta‐analyses [8]. However, *I* ^2^ does not quantify the absolute magnitude of heterogeneity, but only its relative importance in proportional terms, which limits its isolated interpretation [7, 11, 12]. This distinction between inconsistency and absolute heterogeneity has been strongly reaffirmed in recent methodological reflections, which emphasise that *I* ^2^ should not be interpreted as a measure of the absolute amount of between‐study variability [13]. Moreover, its value is strongly influenced by the number of studies and the precision of individual estimates, and it may assume high values even when the true between‐study variance is small, particularly in meta‐analyses with large sample sizes [11]. Consequently, low *I* ^2^ values, including *I* ^2^ = 0%, indicate only the absence of statistically detectable inconsistency and should not be interpreted as evidence of the absence of real variability among true effects [7, 8]. As with Cochran's *Q*, *I* ^2^ requires contextual interpretation in conjunction with other heterogeneity metrics and clinical and methodological considerations, since identical *I* ^2^ values can reflect markedly different absolute effect ranges, meaning that *I* ^2^ alone provides no information on the clinical relevance of the observed variability [6, 7, 8, 9, 11, 14].

Another problem in the interpretation of Higgins' *I* ^2^ statistic concerns the use of the interpretative thresholds proposed in the Cochrane Handbook [6]. These ranges (0%–40%, 30%–60%, 50%–90%, 75%–100%) present explicit overlaps, such that certain values may fall into more than one category, highlighting their non‐dichotomous and inherently imprecise nature [8, 11]. This reinforces that *I* ^2^ should not be used as a rigid decision‐making criterion. Even the developers of the *I* ^2^ index have cautioned against the uncritical application of such thresholds, noting that they were never intended to function as decision rules for pooling, model choice, or interpretation [13].

This interpretative ambiguity underscores the need to complement *I* ^2^ with measures that express the absolute magnitude of heterogeneity, such as *τ* (between‐study standard deviation), as well as with prediction intervals, which translate this variability into clinically interpretable terms [7, 11]. *τ* (between‐study standard deviation) directly quantifies the absolute dispersion of true effects around the pooled mean in a random‐effects model and is expressed on the same scale as the outcome. *τ* ^2^ (between‐study variance) corresponds to the between‐study variance used for model estimation [7]. Unlike *I* ^2^, which describes the relative importance of heterogeneity in proportional terms, *τ* provides a direct and clinically interpretable estimate of the absolute magnitude of between‐study variability [7, 11]. As a parameter, *τ* does not depend on the number of studies or the precision of individual estimates. Its estimate (τ̂), however, is unstable with few studies and varies according to the estimator used. Nevertheless, τ remains conceptually more appropriate than *I* ^2^ for quantifying absolute magnitude, provided the uncertainty of the estimate is acknowledged. Interpretation of *τ* may still require contextualisation, particularly when effect measures are expressed on less intuitive scales (e.g., logarithmic transformations) [9]. For this reason, its usefulness is maximised when interpreted in conjunction with clinically relevant parameters, such as the minimal important difference, and with tools that translate this variability into intervals directly applicable to clinical practice [11, 15]. Recent methodological work has explicitly recommended this shift in emphasis, arguing that *τ* and prediction intervals are more informative than *I* ^2^ for understanding the magnitude and practical implications of heterogeneity [13].

More recently, Yang and colleagues proposed the *I*_*A* ^2^ statistic to quantify standardised population‐level heterogeneity rather than observed effect sizes, thereby reducing the strong influence of study sample size that affects conventional *I* ^2^ [16, 17]. Although conceptually attractive, particularly for continuous outcomes, it remains a relatively new metric with limited application beyond mean differences and standardised mean differences and should be interpreted as a complementary tool rather than as a decision rule.

The prediction interval broadens the interpretation of a meta‐analysis by estimating the plausible range of effects that could be observed in a new study, explicitly incorporating the estimated between‐study variance (*τ* ^2^), which reflects the underlying dispersion of effects across studies [7, 15]. Unlike the confidence interval for the mean effect, which reflects only the uncertainty associated with the estimation of the average effect, the prediction interval considers the full distribution of the underlying true effects and provides a direct estimate of the variability expected in future contexts deemed sufficiently similar to the included studies [6, 15]. In random‐effects models, the prediction interval is derived from the pooled mean effect and the estimated between‐study variance (*τ* ^2^), incorporating uncertainty in both the pooled effect and the between‐study variance estimate, and is typically calculated using a *t* distribution with *k* − 2 degrees of freedom [15]. In this way, the prediction interval offers a more realistic representation of uncertainty and generalisability of the results, particularly in situations of relevant heterogeneity, in which the true effect may vary substantially across different clinical settings [15, 18]. Thus, its use helps to avoid overly optimistic interpretations based solely on the mean effect and supports a more informed assessment of the applicability of meta‐analytic findings to clinical practice [6, 15]. It is important to note, however, that the estimate of *τ* ^2^ and the resulting prediction interval are themselves unstable when the number of studies is small. In such settings, both the absolute magnitude of heterogeneity and the width of the prediction interval should be interpreted with particular caution.

Taken together, these metrics should be viewed as complementary elements within a structured interpretative framework. Figure 1 presents an integrated approach to heterogeneity assessment, illustrating the complementary roles of the *Q* test, *I* ^2^, *I*_*A* ^2^, *τ*, and the prediction interval. The assessment is iterative and context‐dependent rather than sequential, underscoring the need to integrate these indicators with clinical and methodological judgement.

**Figure 1:**
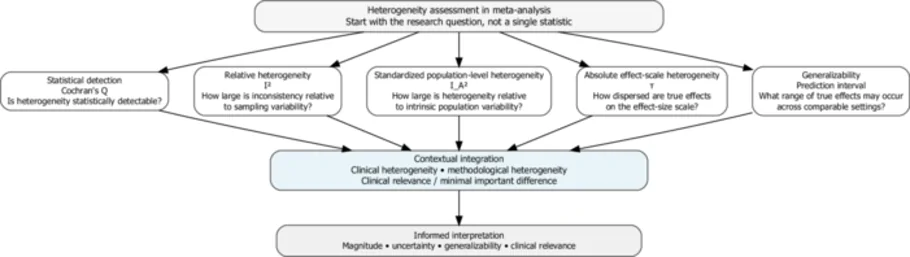
Integrated framework for heterogeneity assessment in meta‐analyses. The framework presents complementary statistical metrics (Cochran's *Q*, *I* ^2^, *I*_*A* ^2^, *τ*, and the prediction interval) that should be interpreted jointly and iteratively, rather than in a fixed sequential order, and integrated with clinical and methodological judgement.

Interpreting heterogeneity in meta‐analyses requires recognition of its multidimensional nature, which cannot be adequately captured by the isolated reading of a single statistical metric. The uncritical use of *I* ^2^ as a decision‐making rule oversimplifies an inherently complex problem and may lead to inappropriate methodological and clinical inferences. Metrics such as the *Q* test, *I* ^2^, *τ* (the between‐study standard deviation), and the prediction interval inform distinct yet complementary dimensions of heterogeneity and should be interpreted in an integrated manner, in light of clinical and methodological considerations and the plausibility of the observed effects [19]. From a clinical perspective, heterogeneity should be evaluated in relation to clinically meaningful effect thresholds, since a wide prediction interval that spans both benefit and harm has fundamentally different implications for decision‐making than one that remains within a clinically trivial range [20].

By recognising that these statistics function as tools to inform interpretation rather than substitutes for scientific judgement, evidence synthesis becomes more transparent, clinically relevant, and methodologically robust. Ultimately, interpreting heterogeneity beyond *I* ^2^ is not merely a statistical refinement, but a prerequisite for producing evidence that meaningfully informs clinical practice and health decision‐making. This perspective aligns with recent reflections emphasising that heterogeneity metrics should support, rather than replace, contextual scientific judgement [13].

Table 1 provides an operational framework illustrating why I² should be interpreted alongside clinical and methodological considerations, *I_A*², τ², and prediction intervals.

**Table 1:** Operational framework for interpreting heterogeneity in meta‐analyses.

Step	Principle	Operational guidance
1	Start with the question, not the statistic	Confirm that included studies address a sufficiently similar PICO; statistical heterogeneity is largely a consequence of underlying clinical and methodological diversity.
2	Treat heterogeneity as multidimensional	Explicitly consider clinical, methodological, and statistical heterogeneity rather than relying on a single quantitative metric.
3	Use Cochran's *Q* for detection only	Cochran's *Q* indicates whether heterogeneity is statistically detectable, but not its magnitude; a non‐significant Q should be interpreted as “not detectable,” not “absent.”
4	Do not use *I* ^2^ as a decision rule	*I* ^2^ reflects relative inconsistency, not absolute variability, and is influenced by the number of studies and their precision.
5	Avoid rigid *I* ^2^ thresholds	Conventional *I* ^2^ categories overlap and are inherently non‐dichotomous; values should be interpreted contextually rather than as fixed cut‐offs.
6	Consider *I*_*A* ^2^ as a standardised population‐level measure	*I*_*A* ^2^ quantifies standardised population‐level heterogeneity independent of sample size and may help identify situations where high *I* ^2^ values are driven primarily by precision rather than clinically meaningful differences (particularly for continuous outcomes).
7	Add τ to quantify absolute heterogeneity	τ estimates the between‐study standard deviation on the effect‐size scale and provides a direct quantification of absolute heterogeneity, in contrast to *I* ^2^, which reflects relative inconsistency.
8	Interpret τ against clinically meaningful anchors	When τ is expressed on less intuitive scales (e.g., log‐transformed effect measures), contextualise its magnitude using clinically relevant benchmarks, such as minimal important differences.
9	Report prediction intervals to communicate generalisability	Prediction intervals describe the plausible range of true effects in a new setting, helping to avoid overly optimistic interpretations based solely on the pooled mean effect.
10	Integrate metrics as complementary tools	Use Cochran's *Q*, *I* ^2^, *I*_*A* ^2^, *τ*, and prediction intervals as complementary components within a structured interpretive framework, rather than as substitutes for contextual scientific judgement.
11	Align conclusions with clinical and methodological plausibility	When heterogeneity is relevant, emphasise limits to applicability and clearly state the conditions under which effects may vary across contexts.

*Note: I* ^2^ reflects the proportion of total variability due to heterogeneity rather than sampling error; *I*_*A* ^2^ provides a sample‐size–independent estimate of standardised population‐level heterogeneity; τ represents the between‐study standard deviation and directly quantifies absolute heterogeneity on the effect‐size scale; and prediction intervals translate this variability into the plausible range of true effects in a new study.

## Ethics Statement

The authors have nothing to report.

## Conflicts of Interest

The authors declare no conflicts of interest.

## Data Availability

Data sharing not applicable to this article as no datasets were generated or analysed during the current study.
